# Prevalence and factors associated with actinic keratosis in 1346 patients attending at a public dermatology service: a cross-sectional study

**DOI:** 10.1590/1516-3180.2024.0178.07042025

**Published:** 2025-10-13

**Authors:** Ademar Schultz, Lara Bourguignon Lopes, Roberta Ribeiro Batista Barbosa

**Affiliations:** I Dermatologist, Department of Public Policies and Local Development, Escola Superior de Ciências da Santa Casa de Misericórdia de Vitória (EMESCAM), Vitória (ES), Brazil; Professor, Department of Medicine, Centro Universitário de Brasília (CEUB), Brasília (DF), Brazil.; II Physiotherapist, MSc Student, Postgraduate Program in Public Policies and Local Development, Escola Superior de Ciências da Santa Casa de Misericórdia de Vitória (EMESCAM), Vitória (ES), Brazil.; III Professor, Department of Public Policies and Local Development, Escola Superior de Ciências da Santa Casa de Misericórdia de Vitória (EMESCAM), Vitória (ES), Brazil.

**Keywords:** Keratosis, actinic., Epidemiology., Skin neoplasms., Health policy., Skin cancer., Prevention of diseases., Health public service.

## Abstract

**BACKGROUND:**

Actinic keratosis is a common preneoplastic dermatosis and is the third most common reason for dermatological consultations. Identifying the associated factors, diagnosis, and early treatment of actinic keratosis are crucial for reducing the risk of developing skin cancer and costs to the healthcare system.

**OBJECTIVE:**

To evaluate the prevalence and factors associated with actinic keratosis in individuals treated at a public dermatology service.

**DESIGN AND SETTING:**

A cross-sectional observational study was conducted with 1346 patients treated by a public dermatology service.

**METHODS:**

The demographic and dermatological characteristics of patients were recorded. The presence of actinic keratosis was determined by clinical dermatological identification.

**RESULTS:**

Most participants were elderly, white, and exposed to the sun without protection or during occupational activity. The evolution time of cutaneous lesions was <1 year in 46.8% of participants. Actinic keratosis was the most common skin lesion, being present in 29.3% of participants. The main approach adopted was cryotherapy. Keratosis was associated with white skin color, elderly age, personal and family history of skin cancer, exposure to the sun without protection at work, and limb involvement. When the associated factors were analyzed using a regression model, skin color and affected body segments were the main predictors of actinic keratosis.

**CONCLUSIONS:**

The prevalence of actinic keratosis was 29.3%, being higher in people with fair skin, more than two affected segments, skin lesions on the limbs, older age, and unprotected sun exposure. These indicators are important for supporting decision-making and contributing to improving public policies.

## INTRODUCTION

 Actinic keratosis is a common preneoplastic dermatosis. In Brazil, actinic keratosis represents the fourth most common dermatological diagnosis, only behind acne, photoaging, and nonmelanoma skin cancer. Among individuals aged > 65 years, actinic keratosis is the primary reason for attending a dermatologist.^1^

 Due to its potential to develop into skin cancer, actinic keratosis is of significant importance, given that skin neoplasia is considered a serious public health problem, with a high incidence and impact on the lives of the affected population.^2^ Some public policies have been created to reduce the incidence of cancer and improve quality of life. However, the considerable challenge and difficulty in accessing diagnostic services, among other problems, weaken these policies.^3^

 Studies on actinic dermatosis in Brazil and specific public policies for skin cancer are scarce. With this, epidemiology has emerged as an important ally, providing local diagnosis and information for the development of more efficient, effective, and equitable management models.^4^

 Cross-sectional studies assist in the critical thinking process aimed at identifying recognition, prevention, and treatment strategies. Considering the importance of deepening studies on actinic keratosis, this study sought to improve discussions on the subject, support decision-making by responsible bodies and the population, and improve public policies. 

## OBJECTIVE

 To evaluate the prevalence and factors associated with actinic keratosis in 1,346 individuals treated at a public dermatology service. 

## METHODS

 This was an epidemiological, observational, cross-sectional study with retrospective data collection approved by the CAAE Research Ethics Committee (51477421200005065) on November 30, 2021. This study was conducted in accordance with the recommendations of *Strengthening the Reporting of Observational Studies in Epidemiology* (STROBE), as described by the STROBE *checklist* to guide the writing of cross-sectional observational studies.^5^

 The sample size was obtained by convenience and comprised all people treated in 2019 at a public dermatology reference center, with 1,409 eligible individuals. The inclusion criteria were as follows: patients of both sexes who were treated at that service in 2019, had medical records, and were aged 18 years or older. Patients with incomplete or illegible medical records were excluded. After applying the aforementioned criteria, 1,346 individuals were included in this study. 

 Data were collected by analyzing medical records written manually by the service team. The following variables were used to characterize the demographic profile: sex, age group, color, history of cancer, family history of skin cancer, sun exposure, and reason for sun exposure. The dermatological profile included clinical diagnosis, number of diagnoses, location, number of affected areas, therapeutic approach, and evolution time of skin lesions. 

 The presence or absence of actinic keratosis (i.e., the dependent variable) was determined through clinical dermatological identification based on the following aspects: lesions with an erythematous background and rough appearance, sometimes with scaling, in regions exposed to the sun.^6^

 Dermoscopic examination was performed as necessary for differential diagnosis, considering actinic keratosis with the following aspects: erythema that forms a pinkish-reddish pseudo-vascular network that surrounds hair follicles; yellowish-white scales; thin wavy vessels that surround the follicles; and follicular openings filled with yellowish keratotic plugs.^6^

 Finally, records regarding the proposed treatment (cryotherapy, surgery, appointment at the service, or referral to another service) were collected to identify the conduct in relation to the dermatological diagnosis. 

 Descriptive analyses of the data were performed using absolute and relative frequencies. Bivariate inferential analysis associating the presence or absence of actinic keratosis with independent variables was performed using the chi-square test. A generalized linear model with a binary logistic link function was used to verify the predictor variables of actinic keratosis and prevalence rates. Variables with P < 0.2 in the bivariate analysis were included in the model. Statistical analysis was performed using IBM SPSS version 27 (IBM Corp., Armonk, NY, USA), and a significance level of 5% (P ≤ 0.05) was adopted for all analyses. 

## RESULTS

 The study population comprised mostly elderly (64.6%), female (60.1%), and white (73.5%) individuals. Of these, 34% had a history of cancer, and 36.4% reported cases of skin cancer in the family. Most participants were exposed to the sun without protection (74.4%), mainly because of work (**Table 1**). 

**Table 1:** Demographic profile of patients treated by a public dermatology service in 2019

**Variables**		**n = 1346 (%)**
**Female sex**		809 (60.1)
**Age range**		
	Adult	467 (34.3)
	Elderly	879 (64.6)
**Skin color**		
	White	989 (73.5)
	Brown/black	357 (26.5)
**Cancer history**		458 (34)
**Family history of skin cancer**		490 (36.4)
**Sun exposure**		
	Did not expose themselves	90 (6.7)
	Unprotected exposure	1002 (74.4)
	Protected exposure	254 (18.9)
**Reason for sun exposure, n = 1256**		
	Leisure	428 (34.1)
	Professional	547 (43.5)
	Both	281 (22.4)

 Dermatological diagnoses and treatment profiles are presented in **Table 2**. The presence of actinic keratosis was recorded in 29.3% of samples, indicating its high frequency in the studied population. Actinic keratosis was the most frequent dermatological diagnosis, followed by basal cell and spinocellular carcinomas. Most lesions were recent (less than a year old); lesions were located in the cephalic segment in more than half of the cases. Surgery, cryotherapy, and guidance stood out among the most widely adopted therapeutic approaches in the studied locations. 

**Table 2:** Dermatological profile and procedures adopted in the face of diagnosis in patients treated by a public dermatology service in 2019

**Variables**		**n = 1346 (%)**
**Clinical diagnosis**		
	Actinic keratosis	395 (29.3)
	Basal cell carcinoma	327 (24.3)
	Squamous cell carcinoma	49 (3.6)
	Melanoma	9 (0.7)
	Other malignant tumors	11 (0.8)
	Other dermatoses	493 (36.6)
	Absence of dermatoses	132 (9.7)
**Location of the injury**		
	Head	797 (59.2)
	Stem	373 (27.7)
	Limbs	620 (46.1)
**Number of affected locations (n = 1345)**		
	One location	956 (71)
	Two locations	307 (22.8)
	Three or more locations	82 (6.1)
**Evolution time of lesions**		
	<1 year	630 (46.8)
	1–3 years	393 (29.2)
	>3 years	323 (24)
**Conduct**		
	Guidance	455 (33.8)
	Surgery	379 (28.2)
	Cryotherapy	294 (21.8)
	Biopsy	35 (2.6)
	Electrocauterization	21 (1.6)
	Formula	19 (1.4)

 Regarding the association between the demographic profile and the presence of actinic keratosis, a statistically significant difference was found in white individuals (P < 0.001) aged > 60 years (P < 0.001) who were exposed to the sun without protection (P = 0.001) for professional reasons and had a personal (P< 0.001) and family (P = 0.003) history of skin cancer (**Table 3**). 

**Table 3:** Association of actinic keratosis with the demographic profile of patients treated by a public dermatology service in 2019

**Variables**		**Actinic keratosis**		
		**No n = 951 (70.7%)**	**Yes n = 395 (29.3%)**	**P**
**Sex**				
	Female	585 (61.5)	224 (56.7)	0.101^a^
	Male	366 (38.5)	171 (43.3)	
**Skin color**				
	White	639 (67.2)	**350 (88.6)^b^**	< 0.001^*^,^a^
	Brown/black	**312 (32.8)^b^**	45 (11.4)	
**Age range**				
	Adults	**376 (39.5)^b^**	91 (23)	< 0.001^*^,^a^
	Elderly	575 (60.5)	**304 (77)^b^**	
**Skin cancer history**		290 (30.5)	**168 (42.5)^b^**	< 0.001^*^,^a^
**Family history of skin cancer**		322 (33.9)	**168 (42.5)^b^**	0.003^*^,^a^
**Exhibition**				
	Without protection	691 (71.6)	**318 (80.5)^b^**	0.001^*^,^a^
	With protection	**196 (20.3)^b^**	61 (15.4)		
	Did not expose themselves	**78 (8.1)^b^**	16 (4.1)		
**Exposure ratio, n = 1256**				
	Leisure	**322 (36.7)^b^**	106(28)	< 0.001^*^,^a^
	Professional	348 (39.7)	**199 (52.5)^b^**		
	Both	207 (23.6)	74 (19.5)		

* P ≤ 0.05

a Pearson’s chi-square

b adjusted residual > 1.96

**Table 4** presents the relationship between actinic keratosis and dermatological profile, revealing the association of actinic keratosis with location on the limbs (P < 0.001) and two or three affected sites (P < 0.001). The most commonly adopted approaches for keratosis were cryotherapy and formula. 

**Table 4:** Association of actinic keratosis with he dermatological profile of patients treated by a public dermatology service in 2019

**Variables**		**Actinic keratosis**		
		**No n = 951 (70.7%)**	**Yes n = 395 (29.3%)**	**P**
**Clinical diagnosis**				
	Melanoma	8 (0.8)	1 (0.3)	0.402^b^,^*^
	Squamous cell carcinoma	**41 (4.3)**	8 (2)	0.041^a^,^*^
	Basal cell carcinoma	**277 (29.1)^c^**	50 (12.7)	< 0.001^*^,^a^
	Other malignant tumors	12 (1.3)	2 (0.5)	0.213^b^,^*^
**Location**				
	Head	554 (58.3)	243 (61.5)	0.267^a^
	Stem	256 (26.9)	117 (29.6)	0.313^a^
	Limbs	369 (38.8)	**251 (63.5)^c^**	< 0.001^*^,^a^
**Number of affected locations (n = 1345)**				
	One location	**746 (78.4)^c^**	210 (53.3)	
	Two locations	165 (17.4)	**142 (36)^c^**	< 0.001^*^,^a^
	Three or more locations	40 (4.2)	**42 (10.7)^c^**	
**Evolution time of lesions**				
	<1 year	450 (47.3)	180 (45.6)	
	1–3 years	263 (27.7)	130 (32.9)	0.119
	>3 years	238 (25)	85 (24)	
**Conduct**				
	Guidance	**409 (43.0)^c^**	46 (11.6)	< 0.001^*^,^a^
	Surgery	267 (28.1)	112 (28.4)	0.918^a^
	Cryotherapy	88 (9.3)	**206 (52.2)^c^**	< 0.001^*^,^a^
	Biopsy	**30 (3.2)**	5 (1.3)	0.047^*^,^a^
	Electrocauterization	13 (1.4)	8 (2)	0.375^a^
	Formula	5 (0.5)	**14 (3.5)^c^**	< 0.001^*^,^a^

* P < 0.05

a Pearson’s chi-square

b Fisher-Freeman exact

b adjusted residual > 1.96

 When the variables were inserted into a regression model to investigate the predictive factors for actinic keratosis (**Table 5**), the prevalence of actinic keratosis was found to be higher in people with fair skin (300%), more than two affected segments (211%), skin lesions on the limbs (186%), older age (177%), and unprotected sun exposure (137%) than in those without keratosis (P < 0.05). 

**Table 5:** Predictor variables associated with actinic keratosis and the respective prevalence ratios according to logistic regression analysis

**Variables**	**P value**	**PR**	**95% CI**	
			**Bottom**	**Higher**
Light skin	< 0.001	3,077	2,167	4,369
Elderly	< 0.001	1,777	1,336	2,365
Unprotected sun exposure	0.043	1,373	1,011	1,866
>2 segments affected	< 0.001	2,119	1,562	2,873
Skin lesions on limbs	< 0.001	1,867	1,407	2,477

PR = prevalence ratio; CI = confidence interval.

## DISCUSSION

 With the global increase in life expectancy, the number of individuals with actinic keratosis is estimated to also increase.^7^ In countries such as Australia, for example, the prevalence is approximately 40%.^8^ In Austria, one study reported a prevalence of 31% in patients aged > 30 years.^9^ In this study, actinic keratosis was present in 29% of the studied sample, approaching global statistics. 

 Skin cancer represents the most common type of cancer in Brazil, with a rate of 223.6 per 100,000 inhabitants.^10^ In 2023, approximately 229,000 cases of skin cancer were detected in the Brazilian population.^10^ Considering that actinic keratosis is a precancerous lesion, the high prevalence found in this study is an important public health problem. 

 Injury is significantly associated with older age and can be explained by the longer period of sun exposure in this population, as age is an independent risk factor for the development of sun-related injuries.^7 ,11^

 Skin neoplasms are also more common in light-skinned individuals, who are inadvertently exposed to the sun during childhood and adolescence.^11^ Our findings corroborate this statement; the white population corresponded to 73.1% of the cases analyzed, with a significant association for the presence of actinic keratosis. 

 Due to aesthetic and cultural demands, women seek health services more frequently and earlier, enabling diagnosis and treatment in the early stages of the disease.^6^ This fact explains the greater frequency of women in the dermatological program, albeit without a statistical association with actinic keratosis. 

 Just over one-third of the population analyzed (33.7%) already had some form of skin cancer. More recent studies have demonstrated that 50% of those who have had a basal cell skin cancer subtype will have another within 5 years after diagnosis.^11^ Regarding the history of skin cancer in the family, there is a tendency for this ratio to increase each year.^1^

 In 2013, the National Policy for Cancer Prevention and Control was established to reduce the incidence of cancer and improve the quality of life of patients with cancer. The policy aims to promote the prevention, early diagnosis, and timely treatment of this condition.^12^ However, there have been great challenges, including the difficulty in accessing diagnostic services due to the availability of services, equipment, and geographic location, as well as the delay in carrying out consultations and examinations necessary for diagnosis.^12^

 Unprotected sun exposure showed a statistically significant association with the presence of actinic keratosis, which may reflect a lack of population guidance regarding the risks of sun exposure. In a study conducted in Mato Grosso do Sul, more than half of the participants never used chemical photoprotection (65%); among those who used chemical photoprotection, 57.1% did not reapply the product during the day.^13^

 Healthcare professionals have the duty to promote health education and communicate with the population through easy and understandable language while envisioning the exchange of information between individuals, respecting the individuality and peculiarities of each one, and enabling health promotion through educational practices. This is the most efficient method to promote health and guide healthy lifestyle practices. This process can be conducted anywhere and at any time, mainly during campaign events, in schools, in environments with greater sun exposure (e.g., beaches and clubs), and in health units through primary care.^14^

 The evolution time of skin lesions was < 1 year in most samples. With respect to actinic keratosis, time is decisive for its progression to invasive squamous cell carcinoma (SCC); the longer the time without treatment, the greater the probability of progression to invasion. At 1 year of evolution, the rate of conversion of lesions to malignancy ranges from approximately 0.25% to 20%; however, some lesions remain stable throughout their evolution, which can last for > 3 years.^15^

 Patients with multiple lesions caused by sun exposure have areas or fields of cancerization and regions of skin that apparently do not have lesions identified as neoplastic or preneoplastic, but are around those with this denomination. Attention should be paid to these areas, as cellular genetic material may have already been modified by UV rays.^16^ This fact draws attention when analyzing **Table 4**. Of the 365 patients who had actinic keratosis, 61 (12.7%) also had other skin lesions, especially basal cell carcinomas. 

 It is known that the risk of developing SCC and basal cell carcinoma is greater than that in the general population.^17^ An international population-based study showed that the probability of patients with actinic keratosis to develop skin cancer was six times higher than that in patients without actinic keratosis.17 However, SCC was present in only 2% of patients, contradicting research that dictates the transformation of actinic keratosis into SCC.^17-19^

 Actinic keratosis is preferentially located in areas exposed to the sun, such as the face, head, neck, neck, shoulders, forearms, and back of the hands, representing 75% of lesions.^20^ These data are consistent with the results of our study, as head injuries accounted for 50% of injuries treated by the service, increasing to 61.5% in the presence of actinic keratosis. 

 Furthermore, injury was significantly associated with upperlimb involvement. The population, especially the rural population, works with hats and shirts, which act as physical protective barriers against UV rays but neglect areas such as arms, forearms, and hands.^21^

 It is important to highlight that Law 4,027 of 2012 guides the provision of sunscreen as personal protective equipment, which is often not carried out by employers.^22^ Intensive sun protection is the first step in controlling the emergence of actinic keratosis; to eliminate the risk of skin cancer, the ultimate goal is to remove all lesions.^23^

 Early diagnosis and treatment are important to minimize disease progression and severity. Patients are recommended to receive therapy that addresses both visible lesions and subclinical damage (field of cancerization). Although more studies are needed, treating cancer in the field is known to improve long-term prognosis, reduce economic burden, and maximize aesthetic results.^23^

 The choice of treatment depends on effectiveness, duration of use, adverse effects, and cost. For this purpose, topical drugs, photodynamic therapy, and ablative procedures are available, including dermabrasion, lasers, and chemical *peels* .^23^ In this study, the main therapies for actinic keratosis were cryotherapy and surgery. Compared to patients without actinic keratosis, cryotherapy and topical formulations were significantly associated with patients with this lesion. 

 The high cost of these treatments is a problem that hinders public policies on access for patients with cancer. Low income, which contributes to low access to preventive and therapeutic measures, is a risk factor associated with the development of these injuries.^24^ In two years, 9.6 million people joined the group of Brazilians living in poverty. Since the beginning of the historical series of the National Household Sample Survey, poverty has never been as high as in 2021.^25^

 Procedures recommended to treat cancer, such as chemical peels, photodynamic therapy, lasers, and even topical 5-fluorouracil (5-FU), are not available in the public health system or supplementary healthcare. The National Commission for the Incorporation of Technologies into the Unified Health System (SUS, in Portuguese) incorporated photodynamic therapy into the SUS for the treatment of basal cell carcinoma without considering actinic keratosis and fields of cancerization.^26^

 In clinical practice, cryotherapy, which involves the application of highly effective liquid nitrogen, is the first therapeutic modality chosen in the face of actinic keratosis, surpassing even surgery.^27^ As it is a painful procedure, the application may not be feasible owing to its non-tolerability, in addition, cryotherapy does not promote the treatment of the field of cancerization, being restricted to circumstantial injuries.^6^

 For home use, 5-FU is the main drug used to treat actinic keratosis. The area of the skin to be treated must not be larger than 500 cm^2^. Despite not being provided by the Unified Health System, it has a low cost, making it accessible to a significant proportion of patients. Although **Table 4** does not specify the combination of treatments, combined therapy is an excellent modality with a greater therapeutic effect and better usefulness in recalcitrant disease. Therefore, future treatment of actinic keratosis is therapy directed at the lesion and therapy directed at the field of cancerization.^28^

 Currently, the biggest barrier to the treatment of skin cancer and pre-cancer in Brazil is related to adequate treatment time, which is crucial for favorable outcomes. When there is negligence, the chances of a cure decrease and the need for invasive procedures and the emergence of metastases increase.^29^ The public service studied, stands out for its simultaneous access to dermatological and plastic surgery services, without the need for long waits in different locations. The presence of community members contributes to the credibility of the program, as they help provide guidance on the signs and symptoms of skin cancer, thus becoming great promoters of the project. 

 Guidance was the primary conduct adopted (34%). Guiding patients about their injuries is fundamental for therapeutic success and prevention. Health education is an important means of increasing healthy behavioral practices as a principle of primary healthcare.^14^ The National Policy for Popular Health Education assists in political strategies and in defining methods for transforming autonomy, participation, and social control, with a focus on popular health education and strengthening popular care practices.^12^

## CONCLUSION

 The prevalence of actinic keratosis in the study population was 29.3%. The presence of this type of lesion was associated with white skin color, elderly age, unprotected sun exposure, and location on the limbs. 

 Considering that actinic keratosis is a precursor lesion of skin cancer, creating specific public policies to address this issue is important, mainly because it is easily preventable, diagnosable, and treatable. There have been great technological advances in this area. However, access to cutting-edge treatment in public services is not a reality for the Brazilian population because of its high value. The main way to avoid spending excessively is to invest in preventive measures. 

 Therefore, enhancing primary care as a gateway for disease prevention, diagnosis, and treatment is important. Health education can strengthen participation and social control by focusing on popular education and care practices. This important tool will assist health professionals in educating them about risk factors, providing guidance on prevention, such as the use of sunscreen, recognizing suspicious lesions, favoring early diagnosis, and qualifying innovative therapies. 

 Specific public policies for skin cancer and its preneoplastic lesions are essential to the protocols of the Unified Health System. The State’s responsibility for the loss of therapeutic opportunities and increase in morbidity and mortality is conditioned by the existence of a universal health service. This reaffirms the principle of universality, which governs the SUS and which, in reality of many users, is often not incorporated. 

## References

[1] Brazilian Society of Dermatology (2018). Orange December.

[2] Costa FB, Weber MB (2004). Evaluation of solar exposure and sun-protection behaviors among university students in the Metropolitan Region of Porto Alegre, Brazil. An Bras Dermatol.

[3] Instituto Nacional do Câncer (2019). Estimativa 2020: incidência de câncer no Brasil.

[4] Roncalli AG (2006). Epidemiology and collective oral health: a shared journey. Ciênc Saúde Coletiva.

[5] Von EE, Altman DG, Egger M (2007). Strengthening the Reporting of Observational Studies in Epidemiology (STROBE) statement: guidelines for reporting observational studies. BMJ.

[6] Reinehr CPH, Bakos RM (2019). Actinic keratoses: review of clinical, dermoscopic, and therapeutic aspects. An Bras Dermatol.

[7] Padilla RS, Connor RF (Ed) (2019). UpToDate.

[8] Frost CA, Green AC (1994). Epidemiology of solar keratoses. Br J Dermatol.

[9] Eder J, Prillinger K, Korn A, Geroldinger A, Trautinger F (2014). Prevalence of actinic keratosis among dermatology outpatients in Austria. Br J Dermatol.

[10] Instituto Nacional de Câncer (2022). Estimativa 2023: incidência de câncer no Brasil.

[11] AC Camargo Cancer Center (2017). Nos homens, o câncer de pele é mais comum nas costas e mais agressivo, alerta pesquisa do A.C.Camargo Cancer Center.

[12] Brasil (2013). Institui a Política Nacional de Prevenção e Controle de Cancer no âmbito do Sistema Único de Saúde. Diário Oficial da União.

[13] Martins VMD (2019). Prevalence of actinic keratosis: evaluation in the population of the Jardim Botânico Basic Health Unit in the municipality of Sinop/Mato Grosso in 2019.

[14] Gueterres ÉC, Rosa EO, Silveira A, Santos WM (2017). Health education in the school context: integrative review study. Enfermería Global.

[15] Callen JP, Bickers DR, Moy RL (1997). Actinic keratoses. J Am Acad Dermatol.

[16] Willenbrink TJ, Ruiz ES, Cornejo CM (2020). Field cancerization: Definition, epidemiology, risk factors, and outcomes. J Am Acad Dermatol.

[17] Chen GJ, Feldman SR, Williford PM (2005). Clinical diagnosis of actinic keratosis identifies an elderly population at high risk of developing skin cancer. Dermatol Surg.

[18] Glogau RG (2000). The risk of progression to invasive disease. J Am Acad Dermatol.

[19] Moon TE, Levine N, Cartmel B (1997). Effect of retinol in preventing squamous cell skin cancer in moderate-risk subjects: a randomized, double-blind, controlled trial. Southwest Skin Cancer Prevention Study Group.

[20] Smit P, Plomp E, Neumann HA, Thio HB (2013). The influence of the location of the lesion on the absolute risk of the development of skin cancer in a patient with actinic keratosis. J Eur Acad Dermatol Venereol.

[21] Cezar-Vaz MR, Bonow CA, Piexak DR (2015). Skin cancer in rural workers: knowledge and nursing intervention. Rev Esc Enferm USP.

[22] Moura PF, Oliveira CSP, Oliveira CF, Miguel MD (2016). Skin cancer: a public health issue. Visão Acadêmica.

[23] Wennberg AM, Stenquist B, Stockfleth E (2008). Photodynamic therapy with methyl aminolevulinate for prevention of new skin lesions in transplant recipients: a randomized study. Transplantation.

[24] Mesquita LG, Diniz SF, Queiroz FTH (2020). Skin Cancer and Family Income: an Ecological Study. Rev Bras Cancerol.

[25] Mapa da nova pobreza: Estudo revela que 29,6% dos brasileiros têm renda familiar inferior a R$ 497 mensais.

[26] Usuários do SUS com câncer de pele basocelular serão tratados com inovação 100% nacional.

[27] Thai KE, Fergin P, Freeman M (2004). A prospective study of the use of cryosurgery for the treatment of actinic keratoses. Int J Dermatol.

[28] Skin Cancer Foundation (2022). Skin cancer in people of color: what you need to know.

[29] Finger B, Limberger T (2019). Acesso ao tratamento oncológico no SUS: a responsabilidade civil do Estado pela perda da chance de cura ou de sobrevida das pacientes com câncer de mama. Rev Dir Gar Fund.

